# The Effects of Prescribed Fire on Artificial Wild Turkey Nest Survival in Closed‐Canopy Mixed Hardwood Forest

**DOI:** 10.1002/ece3.71410

**Published:** 2025-05-07

**Authors:** Mariah G. McInnis, Robert A. Gitzen, Bret A. Collier, William D. Gulsby

**Affiliations:** ^1^ School of Forestry and Wildlife Sciences Auburn University Auburn Alabama USA; ^2^ School of Renewable Natural Resources Louisiana State University Agricultural Center Baton Rouge Louisiana USA

**Keywords:** artificial nest, canopy cover, *Meleagris gallopavo*, prescribed fire, wild turkey

## Abstract

The eastern wild turkey ( *Meleagris gallopavo* ) is an economically and culturally important upland game bird that has recently declined in abundance across portions of the Southeast. Prescribed fire can be used to improve vegetation conditions for wild turkey nesting and brooding, but there are concerns that the application of large‐scale prescribed fire can directly or indirectly impact turkey nest success. Therefore, there is a need to improve understanding of the effects of large‐scale burns on turkey reproduction, particularly how fire effects on vegetation might affect nest success rates. We implemented an artificial nest study on the Talladega National Forest in northeast Alabama, where prescribed fire is implemented across ≤ 8000 ha annually in large (> 300 ha) burn units. We monitored a total of 230 artificial turkey nests during April–May 2019 and 2020. Nests were systematically distributed throughout the study area at a density of 1 nest/202 ha in areas burned 1, 2, 3, 4, and 5–10 years prior to ensure proportional representation of time since fire. The overall artificial nest predation rate was 25%. Top predators included gray foxes ( *Urocyon cinereoargenteus* ; 10 nests), opossums ( *Didelphis virginiana* ; 9 nests), and coyotes ( *Canis latrans* ; 7 nests). We did not detect a relationship between time since fire (*p* > 0.05) or vegetation measurements (*p* > 0.05) and artificial nest predation. We believe the patterns we observed were explained by high overstory canopy cover (~90%) across the study area that limited vegetation response to fire. By mediating the potential effects of fire on understory vegetation structure, overstory canopy cover influences the degree to which fire alters concealment cover for nesting hens. Additional research is needed to determine whether large‐scale prescribed fire directly or indirectly affects wild turkey nest success in systems with lower canopy cover. Additionally, our study outlines evidence that vegetation responses to prescribed fire are site‐dependent.

## Graphical abstract

The eastern wild turkey has declined in portions of the Southeast, raising concerns about how large‐scale prescribed fires might affect nest success. To explore this, we conducted an artificial nest study in Alabama's Talladega National Forest, where prescribed fires cover large burn units annually. Our findings suggest no relationship between time since fire, vegetation, and nest predation, likely due to high overstory canopy cover (~90%) limiting vegetation response to fire. Further research is needed to understand fire's impact on turkey reproduction in areas with lower canopy cover.

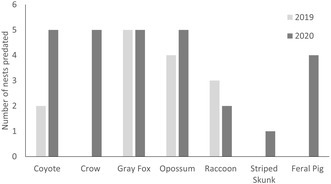

## 1 Background

The eastern wild turkey ( *Meleagris gallopavo silvestris* ; hereafter, wild turkeys) is one of the most economically and culturally important game species in the United States (Pollentier et al. 2014). For example, in Alabama, an estimated $45 million is spent annually on spring wild turkey hunting (Barnett and Barnett 2008), and land managers place high importance on maintaining abundant wild turkey populations. However, Tapley et al. (2011) noted that wild turkey populations and spring and fall hunter harvests began to decline in some areas during the early 2000s, and Ericksen et al. (2015) reported similar findings more recently, which has caused concern among hunters and land managers. Declines could be due to a lack of nesting and brood‐rearing cover (Thogmartin 1998; Wood et al. 2019; Chamberlain et al. 2020), as evidenced by region‐wide declines in wild turkey productivity (Byrne et al. 2015). Therefore, understanding how management practices such as prescribed fire influence wild turkey habitat quality is important for managers across the Southeast.

Prescribed fire generally reduces woody plant coverage and increases herbaceous plants in the understory (Sisson et al. 1990; Sparks et al. 1998; Jones et al. 2002; Wood et al. 2018). However, fire frequency plays an important role in shaping understory plant community composition. Specifically, annual and biennial burning typically result in an herbaceous‐dominated understory, whereas triennial burning is associated with a shift to woody plant dominance (Glitzenstein et al. 2012). Wild turkey use of burned areas reflects these changes (Cohen et al. 2019). Adult female wild turkeys select for recently burned areas and avoid those not burned in the past two years in upland pine (*Pinus* spp.) systems (Martin et al. 2012), and adult females prefer to nest and brood in pine stands burned 2 years prior (Yeldell, Cohen, Little, et al. 2017). Regular application of fire maintains understory vegetation at a level that conceals the female and her nest, while affording the female adequate visibility to detect predators (Wilson 2005). Additionally, Jones et al. (2004) reported that raccoons ( *Procyon lotor* ), which are major turkey nest predators, selected unburned over burned stands, and Boone et al. (2024) found that raccoons were negatively associated with increased burn frequency. Fire can also improve brooding cover by facilitating movement of the female and her brood and increasing abundance of insects, which are important in the poult diet (Little et al. 2014; McCord et al. 2014). In pine systems, brooding females still select for areas burned 0–2 years prior during the day but prefer areas burned 3–6 years prior for roosting at night (Wood et al. 2019). However, in areas with a typical post‐fire dynamic, it is likely that wild turkeys use the edges of areas burned less than one year prior to reduce predation risk until the understory provides substantial visual concealment (Kilburg et al. 2015; Yeldell, Cohen, Prebyl, et al. 2017; Cohen et al. 2019).

Although fire has many positive benefits for wild turkeys, there are still concerns among wild turkey managers and hunters about the effects of burning during the early growing season (April–May), which coincides with the turkey nesting season. There is little evidence that wild turkey nests are impacted by nesting‐season fire, likely because females often nest in recently burned stands not scheduled for burning in the current nesting season (Yeldell, Cohen, Little, et al. 2017; Cohen et al. 2019; Wann et al. 2020). For example, on a North Carolina site where fire was generally implemented every three years during March–June, only 3.3% of monitored nests were destroyed by fire, and no more than 6% of all turkey nests were exposed to fire annually (Kilburg et al. 2014). Although that study site was burned every three years, which normally provides adequate nesting cover in upland pine systems, Kilburg et al. (2014) noted that the combination of low‐productivity soils and growing season burns may have reduced nesting cover to the point that it was less preferred for nesting. Nonetheless, females can re‐nest if their nests are destroyed by fire (Moore et al. 2010), though some studies have shown that nest success decreases for subsequent nests (Badyaev 1995; Byrne and Chamberlain 2013). Overall, it appears that the direct impact of fire on turkey nests is limited. If only a small number of nests are lost, it is thought that the overall habitat benefits likely outweigh the costs (Kilburg et al. 2014; Jones et al. 2002).

Although direct negative impacts of fire may be low, indirect effects on reproduction should also be considered. For example, fire‐dependent vegetation changes may influence nest and brood success. Females generally choose to nest in stands burned 2 years prior (but soils and moisture regime influence vegetation responses to fire) because of the desirable vegetation conditions they provide (Seiss et al. 1990; Yeldell, Cohen, Little, et al. 2017; Wood et al. 2018). Dreibelbis et al. (2016) found that only 9% of Rio Grande (*M. g. intermedia*) wild turkey females in their study nested in areas that had not been burned during the previous 10 years. By contrast, Pittman and Krementz (2016) reported greater nest success in unburned (36.4%) versus burned stands (14.6%), likely because their site was dominated by closed‐canopy hardwood forests, which limited available sunlight to elicit an understory response post‐fire. Similarly, Kilburg et al. (2014) found greater nest survival in unburned lowlands (60%) compared to burned uplands (10%), probably because the combination of poor soils and frequent fire in the uplands limited understory nesting cover. However, Jones et al. (2002) detected no difference in artificial nest success (i.e., no damaged, missing, or disturbed eggs after 7 days) between areas burned on a two‐year rotation and those left unburned. Clearly, the indirect effects of prescribed burning on nest success are site‐specific, or perhaps a consistent trend in the literature is not apparent due to variation in how researchers categorize time since fire.

The scale of fire may also affect nest success. For example, Pittman and Krementz (2016) found that nest success was greater in unburned versus burned units in an area where fire was implemented at the landscape scale (> 10,000 ha), with average burn units > 1000 ha. The reason for their finding was unclear, as the vegetation characteristics they measured did not differ between successful and unsuccessful nests (Pittman and Krementz 2016). More recently, Cohen et al. (2019) reported that turkeys were less likely to use the interiors of burned areas and recommended that interior areas of burned stands should be < 250 m from adjacent unburned stands. Similarly, Sullivan et al. (2020) found that burn units 23 ha in size received the most use by turkeys and suggested fires should not exceed 200 ha in areas where wild turkeys are a management concern. Although data on selection or avoidance of areas during the breeding season provide some insight as to their suitability for nesting, it is still unclear how the scale of fire translates into the probability of nest success.

Due to the uncertainty regarding the indirect effects of burning during the nesting season or at large spatial scales on nest success, we implemented a study to document nest success rates in an area where burns were conducted on a much larger than average scale (> 1000 ha) using artificial nests systematically distributed throughout the area. Because of the well‐known limitations of artificial nests, our objective was not to quantify expected rates of actual wild turkey nest predation, but rather to document relative differences in nest predation among areas with variable time since fire. Additionally, the strength of using artificial nests for this experiment was that they allowed standardized placement by time since fire, which afforded a level of rigor and replication that cannot be done with real animals. We hypothesized that the effects of prescribed fires on artificial nest predation would be driven mainly by post‐fire changes in vegetation structure, with changes in predator activity also potentially contributing. We predicted that artificial nest predation would be greatest 1–2 years post‐burn due to reduced visual obstruction, lowest 3–4 years post‐burn, and moderate 5–10 years post‐burn.

## 2 Methods

**FIGURE 1:**
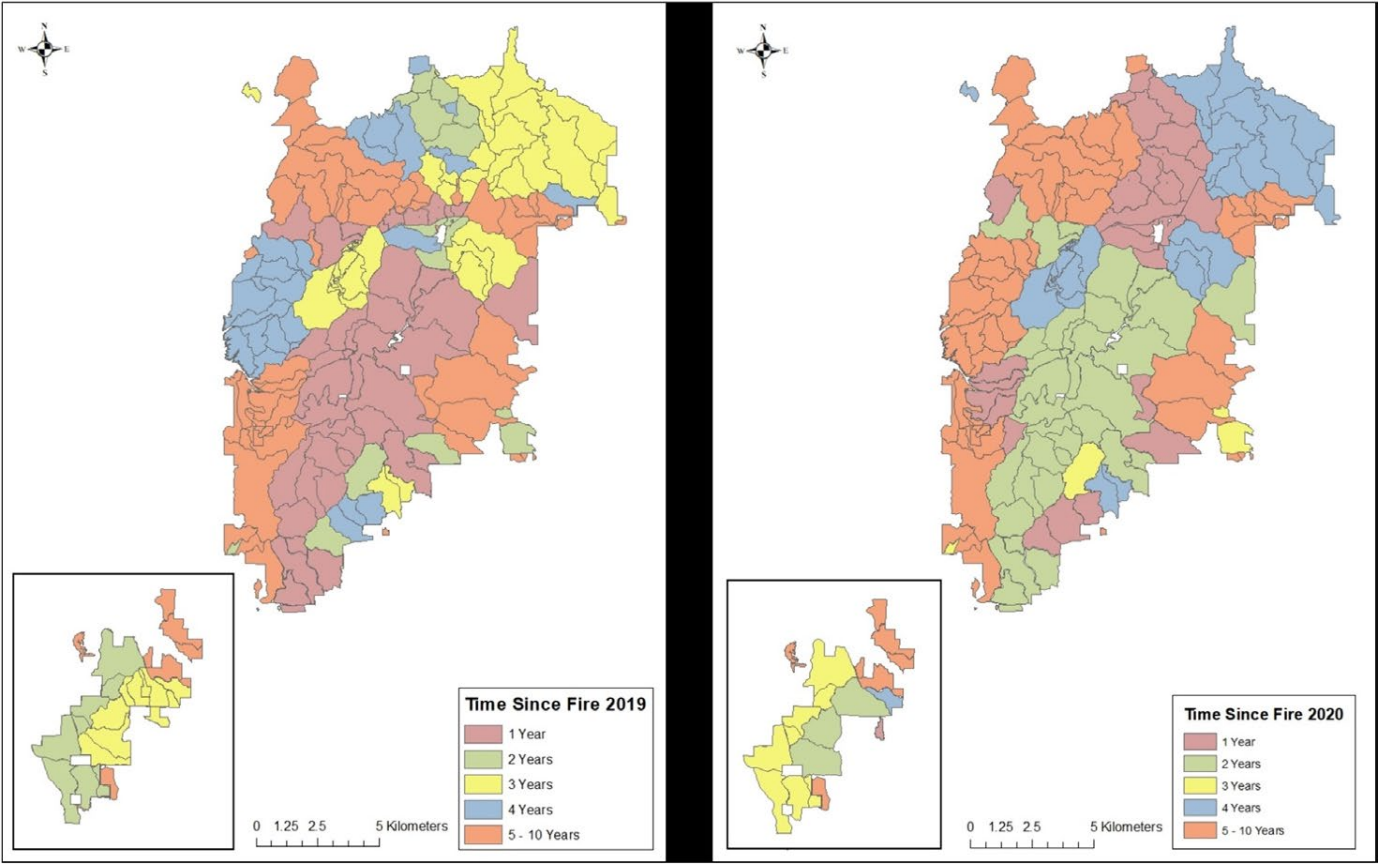
Location of study area in the Shoal Creek district of the Talladega National Forest, Alabama, USA where we examined the relationship between time since fire and nest predation on artificial wild turkey ( *Meleagris gallopavo* ) nests during 2019 and 2020.

We conducted our study on the Shoal Creek Ranger District of the Talladega National Forest in Calhoun and Cleburne counties, Alabama (Figure 1). The study area encompassed approximately 48,000 ha in the Ridge and Valley physiographic region and was managed by the U.S. Forest Service. The topography was steep, and elevation ranged from 200 to 730 m (Womack and Carter 2011). The study area was composed of a variety of forest types, including 37% Oak‐Hickory (*Quercus* spp., *Carya* spp.), 26% loblolly pine ( *Pinus taeda* ), 13% longleaf pine ( *P. palustris* ), and 11% mixed pine‐oak. The climate was subtropical, with a mean annual temperature of 16°C and a mean annual precipitation of 135 cm (Runkle et al. 2017). Soils in the study area were well‐drained and consisted primarily of the Tatum‐Tallapoosa‐Fruithurst association (NRCS 2020). Prescribed fire was used to maintain habitat for the endangered red‐cockaded woodpecker ( *Leuconotopicus borealis* ), restore longleaf pine forest understories, and reduce wildfire fuels. The average fire return interval across the Talladega National Forest was 5–6.5 years. About 16% (7500 ha) of the area was burned on a rotation < 3.5 years, and about 33% of the forest received no fire management. Due to the adjacency of burn units, contiguous burned areas ranged in size from 199 to 5488 ha and averaged 1295 ha. On average, 60% of fires were applied during the dormant season (Jan–Mar; Stober et al. 2020).

### 2.1 Artificial Nests

We systematically distributed 115 artificial nests across the study area during April–May of 2019 and 117 nests during April–May 2020. During 2020, artificial nest sites were located ≥ 100 m from their location during 2019 to prevent bias from predators revisiting the previous year's nest locations. The timing of nest placement was consistent with first nesting attempts of wild turkeys in the Southeast (Moore et al. 2010; Pittman and Krementz 2016). We categorized our study area into areas burned 1, 2, 3, 4, and 5–10 years prior. We then established artificial nests at a density of approximately 1 per 202 ha within each burn class to ensure proportional representation. Specifically, we used the create random points function in ArcGISv10.6 (ESRI Inc., Redmond, WA) to establish potential nest sites prior to conducting field work. All potential nest sites were ≥ 500 m apart and located > 250 m from roads in 2019. We reduced the maximum distance between nests and roads to < 250 m during 2020 to reduce logistical complexity.

We placed each nest ≤ 50 m from the selected point, choosing a nest site with vegetation that would provide visual obstruction from nest predators when available (Fleming and Porter 2015). Nests consisted of three unwashed chicken eggs placed in a slight depression in the ground, using leaves and litter to create a nest bowl. In 2019, we covered each nest with a small patch of burlap around 15 cm in diameter to provide visual concealment, which is typically provided by a female sitting on the nest for a majority of the day (Lohr et al. 2020). Because nest predation rates during 2019 were low compared to those reported in the literature (compared to both natural and artificial nests), we chose not to use burlap during 2020. We wore rubber boots and gloves to minimize human scent around the nest (Pharris and Goetz 1980; Melville et al. 2014). We placed a camera trap 1.5–2 m from each nest at a height of 0.5–1 m, depending on the topography of the location. We used Stealth Cam DS4K (GSM Outdoors, Irving, TX) and Moultrie D‐80 White Flash (Moultrie Products, Birmingham, AL) camera traps. Cameras were oriented north or south to avoid glare from the sun. We set each camera to its greatest motion‐detecting sensitivity and retrieved it after a 2‐week period to prevent bias associated with the scent of decomposing eggs or revisitation of the site to change the eggs (Melville 2019). We deployed an average of 55 nests/week.

### 2.2 Data Collection

We recorded vegetation measurements at each nest site, including visual obstruction, percent cover of plants by functional group, and percent canopy cover, upon returning to collect the camera traps. We estimated visual obstruction using a vegetation profile board (Nudds 1977) placed at the center of the nest bowl and viewed from a distance of 15 m and an observer height of 0.5 m. We recorded visual obstruction in all cardinal directions and averaged the readings to provide one value for the nest site (Byrne et al. 2013). We measured visual obstruction for each of the six 0.5‐m strata on the board on a scale of 1–5, where 1 = 0%–20%, 2 = 21%–40%, 3 = 41%–60%, 4 = 61%–80%, and 5 = 81%–100%. We used a 1 m^2^ Daubenmire frame (Daubenmire 1959) to visually estimate percent cover of grasses, forbs, woody vegetation, and debris or bare ground. We estimated ground cover at the nest bowl location and 15 m in each cardinal direction and averaged the estimates to provide one value per nest. We estimated overstory canopy cover using a spherical densiometer (Lemmon 1956) directly over the nest bowl and 15 m from the nest in each cardinal direction and averaged those values as well.

### 2.3 Scent Stations

We distributed scent stations across the study area during May of each year to estimate the relative abundance of predators. We used the create random points function in ArcGIS to create 108 station locations during each year of the study. Scent stations were placed ≥ 0.3‐km apart and adjacent to secondary roads and trails as much as possible, as predators tend to utilize roads for travel. Once at a point, we would find a nearby trail, road, or cleared area and a corresponding tree where we could place the camera facing either north or south. We then placed a fatty acid scent tablet (Wildlife Control Supplies, East Granby, CT) on the ground 1.5–2 m in front of the camera to act as an attractant. Locations were baited with scent tablets and left for two nights in 2019, but due to low detection rates, we increased the duration to four nights in 2020. Cameras were set on time‐lapse to take a photo every 2 min due to likely differences in detectability between larger (e.g., coyote; *Canis latrans* ) and smaller (e.g., American Crow; *Corvus brachyrhynchos* ) nest predators.

### 2.4 Analysis

We used logistic regression in R (v. 4.0.0, R Core Team 2020) to examine the effects of vegetation measurements on nest predation (predated/not predated). Specifically, we examined a set of models including all combinations of time since fire, visual obstruction from 0 to 1 m, canopy cover, herbaceous ground cover, and woody ground cover as predictor variables, as well as a null model without any of these variables. We used year as a fixed effect in all models. We combined the 0–0.5 m and 0.5–1 m strata because vegetation at these heights is needed for concealment of the nest without hindering a female's ability to detect predators (McCord et al. 2014). We used R package MuMIn (Barton 2009) to rank models by Akaike's information criterion adjusted for small sample sizes (AICc). We also used logistic regression to examine nest predation as a function of distance to, and density of, roads within a 200 m buffer of the nest location. We used analysis of variance (ANOVA) to examine the relationship between vegetation characteristics and time since fire, with Tukey's Honest Significant Difference tests used to compare characteristics among time‐since‐fire groups when an overall effect was detected. We set *α* = 0.05 for all statistical tests.

## 3 Results

We monitored a total of 230 artificial nests during April–May 2019 and 2020 and observed an overall predation rate of 25% (58 nests). The nest predation rate was 16% (18 nests) in 2019 when nests were covered with burlap, and 35% (40 nests) in 2020 when nests were uncovered. Species responsible for nest predation included gray foxes ( *Urocyon cinereoargenteus* ; 10 nests), opossums ( *Didelphis virginiana* ; 9 nests), coyotes (7 nests), raccoons (5 nests), American crows (5 nests), wild pigs ( *Sus scrofa* ; 4 nests), and striped skunks ( *Mephitis mephitis* ; 1 nest). We observed 2 instances of secondary predation (eating ≥ 1 egg after the nest had been predated by another species) by armadillos ( *Dasypus novemcinctus* ).

**FIGURE 2:**
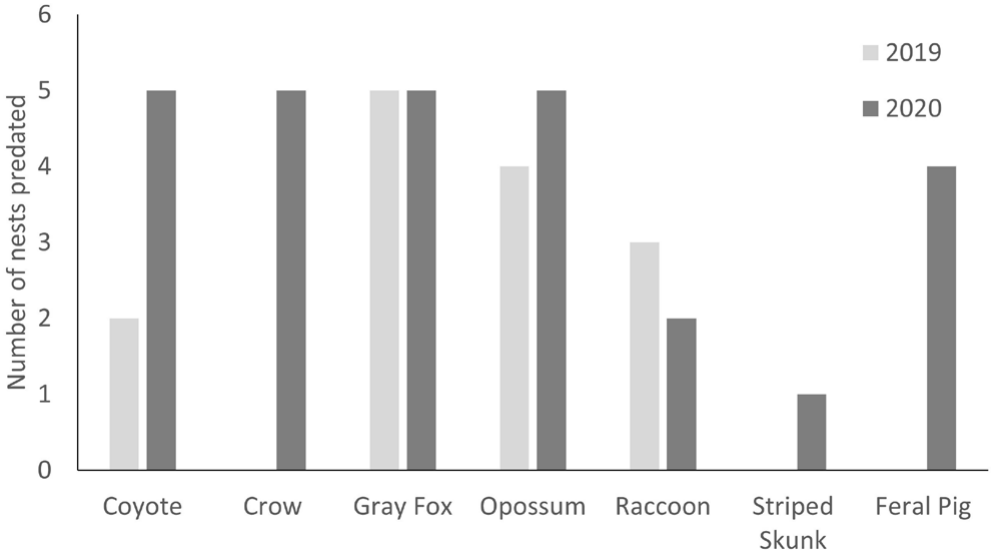
Species‐specific artificial wild turkey ( *Meleagris gallopavo* ) nest predation counts on the Shoal Creek district of the Talladega National Forest, Alabama, USA. Eggs within each nest were covered with unscented burlap cloth during 2019, but not during 2020.

All 5 instances of corvid predation were during 2020, when nests were uncovered (Figure 2). We also documented nest predation by striped skunks (1 nest) and feral pigs (4 nests) during 2020. Other differences between years included an increase in coyote predation from 2019 (2 nests) to 2020 (5 nests), an increase in opossum predation from 2019 (4 nests) to 2020 (5 nests), and a decrease in raccoon predation from 2019 (3 nests) to 2020 (2 nests). Gray fox predation was the same between years. We were unable to identify the species responsible for depredating 17 nests due to camera malfunction. Average time to nest predation was 6.4 days and ranged from 1 to 14 days. We included year as a fixed effect in all of our models predicting the effects of vegetation on nest predation because there was a significant effect of year on this parameter, with depredation 2.91 (95% CI: 1.57–5.59; *p* = 0.002) times as likely in 2020, likely due to the use of burlap coverings in 2019. The null model was the most informative from our candidate set (Table 1). Therefore, we did not interpret parameter estimates for the covariates included in any other models.

**TABLE 1:** AICc model selection for models describing effects of visual concealment at the 0–1 m strata, canopy cover, herbaceous cover, and woody cover on artificial turkey nest success in the Shoal Creek district of the Talladega National Forest, Alabama, USA during 2019 and 2020. Only models with ∆AIC < 4.0 are presented.

Model	DF	logLik	AICc	∆AIC	Weight
Null	2	−123.764	251.6	0.00	0.133
Visual concealment	3	−123.177	252.5	0.88	0.086
Visual concealment + Woody cover	4	−122.357	252.9	1.31	0.069
Woody cover	3	−123.513	253.1	1.55	0.061
Canopy cover	3	−123.538	253.2	1.6	0.006
Canopy cover + Visual concealment	4	−122.659	253.5	1.92	0.051
Herbaceous cover	3	−123.751	253.6	2.03	0.049
Canopy cover + Visual concealment + Woody cover	5	−121.812	253.9	2.31	0.048
Herbaceous cover + Visual concealment	4	−123.12	254.4	2.84	0.042
Herbaceous cover + Visual concealment + Woody cover	5	−122.19	254.6	3.07	0.032
Canopy cover + Woody cover	4	−123.324	254.8	3.24	0.032
Herbaceous cover + Woody cover	4	−123.484	255.1	3.56	0.029
Canopy cover + Herbaceous cover	4	−123.526	255.2	3.65	0.027
Canopy cover + Herbaceous cover + Visual concealment	5	−122.651	255.6	3.99	0.026

Canopy cover averaged 92% across our study site. We did not detect a relationship between time since fire and woody cover in the understory (*F*_4,223_ = 0.9, *p* = 0.45). We detected an effect of time since fire on visual obstruction from 0 to 1 m (*F*_4,223_ = 3.5, *p* = 0.008), and Tukey's post hoc test suggested visual obstruction was greater in areas burned 2 years prior versus 4 years prior (Table 2; *p* = 0.035). We also detected a relationship between time since fire and herbaceous cover (*F*_4,223_ = 3.4, *p* = 0.01), with herbaceous cover generally declining as time since fire increased (Table 2). However, Tukey post hoc tests did not detect significant differences among groups. We detected ≥ 1 species of nest predator at 25% (27/107) of scent stations in 2019 and 43% (45/106) in 2020. Species included coyotes (*n =* 34), gray foxes (*n =* 19), raccoons (*n =* 10), armadillos (*n =* 3), opossums (*n =* 3), bobcats (*n =* 2), and wild pigs (*n =* 1).

**TABLE 2:** Average vegetation measurements and standard errors (SE) of canopy cover (%), visual concealment at the 0–1 m strata (%), herbaceous ground cover (%), and woody cover (%) taken at artificial nest sites varying by time since fire in the Shoal Creek District within the Talladega National Forest AL during 2019 and 2020.

Time since fire	Canopy cover (%)	Visual concealment 0–1 m (%)	Herbaceous cover (%)	Woody cover (%)	% Depredated
1 year	92.01 (1.20)	54 (0.12)	14.45 (1.94)	23.44 (1.52)	21
2 year	91.05 (1.55)	62 (0.12)	11.60 (1.52)	22.81 (1.70)	33
3 year	89.64 (2.83)	52 (0.19)	13.46 (2.90)	21.85 (2.38)	14
4 year	91.70 (2.64)	50 (0.15)	7.17 (1.75)	17.66 (2.01)	12
5–10 years	94.29 (1.24)	60 (0.11)	7.26 (1.02)	20.53 (1.52)	21

## 4 Discussion

Based on previous studies (Badyaev 1995; Streich et al. 2015), we expected artificial nest predation to be greater during years 1–2 post‐burn, decrease in years 3–4 post‐burn, and increase again in years 5–10 post‐burn due to vegetation conditions associated with each time period. However, we did not detect a relationship between time since fire and artificial nest predation, likely due to limited or inconsistent vegetation responses to prescribed fire in our study area. Specifically, although visual obstruction and herbaceous ground cover were related to time since fire, post hoc tests did not indicate a consistent trend. Therefore, it is not surprising that artificial nest predation did not differ by time since fire.

We believe one of the driving forces behind our lack of vegetation response was related to high canopy cover (~90%) across the study area. Jackson et al. (2007) found that the application of prescribed fire alone did not result in adequate turkey nesting cover within closed‐canopy mixed hardwood stands. Others have reported that overall vegetation coverage and response to prescribed fire are lower in areas where sunlight is limiting (Lashley et al. 2011; Keene et al. 2021). Conversely, when prescribed fire is coupled with canopy reduction, vegetation responses are much greater. For example, Pack et al. (1988) saw an increase in brooding cover by the third growing season on sites that were thinned before being burned. Additionally, Byrne and Chamberlain (2013) reported that in a bottomland hardwood forest with 85% canopy cover, turkey nests were often associated with small breaks in the canopy, which they attributed to greater understory plant cover due to increased sunlight. Byrne and Chamberlain (2013) also found that nesting and nest success rates were greater following a hurricane that reduced canopy cover by 30%. Streich et al. (2015) found that for every 5% increase in canopy cover, nests were 1.12 times less likely to occur. At their study site in Georgia, the average percent canopy closure was 54% at nest sites compared to 65% at random locations.

Although canopy cover may have reduced variation in vegetation due to time since fire during our study, the literature suggests conditions may have been conducive to successful nesting. Specifically, woody cover averaged 24% and visual obstruction ≤ 1‐m tall averaged 55% at our nest sites. Others have reported successful nests were in areas with 57–100% visual obstruction ≤ 1‐m tall (Badyaev 1995; Moore et al. 2010), the low end of which is only slightly greater than the average across our sites. Streich et al. (2015) reported that nest sites had an average woody cover of 24%, the same as our average, compared to 12% at random locations. However, several have found little or no support for a link between nest success and vegetation conditions around the nest site (Thogmartin 1999; Fuller et al. 2013; Little et al. 2016; Lohr et al. 2020), which could mean that other factors, perhaps at a larger scale, are more important in predicting nest success (Crawford et al. 2021).

Our overall rate of predation (25%) was low compared to other artificial nest studies. For example, Melville et al. (2014) recorded a predation rate of 61% in East Texas, and Jones et al. (2010) recorded a predation rate of 72% in South Georgia. Our artificial nest predation rates were also lower than those reported for actual nests in the Southeast, which range from 60% to 80% (Byrne et al. 2013; Little et al. 2014; Pittman and Krementz 2016; Wood et al. 2019; Chamberlain et al. 2020). Artificial nest studies often overestimate predation rates (Major and Kendal 1996; Dreibelbis et al. 2011), so the low predation rate for artificial nests in our study may indicate unusually low predator abundance in the study area. Specifically, our scent‐station data showed only a 34% visitation rate by all predators. Gabor et al. (1994) reported a scent‐station visitation rate of 41% just by raccoons in Tennessee. In contrast, raccoons visited only 14% of our scent stations, which may have been driven by the lack of bottomland cover types on our study area preferred by raccoons during spring (Byrne and Chamberlain 2015). However, it is also possible that the time of year influenced our visitation rates, as others have noted significantly lower visitation rates in spring versus fall (Conner et al. 1983).

There were two primary advantages to using artificial nests in our study. One benefit was that it allowed us to determine species‐specific nest predation rates using cameras, which helped solve issues faced in previous nest success studies (Major and Kendal 1996). For example, some previous studies have used quail eggs, which lead to increased small mammal predation (Hartley and Hunter 1998), or relied on tooth impressions in wax eggs, which are error‐prone (Jones et al. 2010). Concerns regarding female disturbance and nest abandonment often preclude researchers from establishing species‐specific predation rates in studies involving natural turkey nests. Another benefit of using artificial nests was that it allowed systematic nest placement by time since fire to rigorously test hypotheses related to environmental factors influencing nest success. Overall, monitoring natural female nests provides more realistic data on predation rates and nest site selection, whereas artificial nests lend themselves to more robust experimental frameworks and documenting species‐specific predation rates.

The species‐specific artificial nest predation rates we observed were similar to some in the literature, but quite different from others. It is important to consider that artificial nests lack the visual and olfactory cues provided by a hen on a real nest, which may influence our results. Conversely, hens only visit the nest briefly each day during the laying period, but do not incubate until all the eggs are laid. Species‐specific predation counts varied between years, probably because nests were covered in 2019 but not in 2020. For example, both coyote and opossum nest predation counts increased from 2019 to 2020, and we only documented nest predation by crows, striped skunks, and feral pigs during 2020. However, year‐to‐year changes in species‐specific predation rates were small (i.e., differed by 1–2 nests), and likely within the range of natural annual variation one might expect. Nest predation by corvids is common for uncovered artificial nests due to the lack of visual concealment of the eggs (Santisteban et al. 2002). We also recorded more incidences of gray fox ( *Urocyon cinereoargenteus* ), coyote ( *Canis latrans* ), and wild pig ( *Sus scrofa* ) predation than other artificial nest studies in the Southeast. However, our wild pig predation rate (2% of nests) was much lower than an artificial nest study in Texas, where pigs depredated 29% of nests monitored with cameras when located within the wild pig's core range (Sanders et al. 2020). We recorded a mesomammal predation rate of 26%, whereas Jones et al. (2010) reported that the artificial nest predation rate by mesomammals in South Georgia was 15%, and Leimgruber et al. (1994) reported an artificial nest predation rate of 20% in Virginia, with striped skunks and raccoons being the most frequently observed predators. Outside the Southeast, Melville et al. (2014) reported an artificial turkey nest predation rate of 61% in East Texas (Melville et al. 2014), with raccoons and opossums being the most common predators. Differences in nest predation rates among studies are likely driven by a variety of factors including methodology, landscape context, predator community composition, and predator abundance.

## 5 Conclusions

Our overall objective was to determine whether large‐scale application of prescribed fire indirectly affected artificial turkey nest success by altering vegetation conditions. Although we found no evidence of an effect of time since fire on artificial nest predation rates, others have reported that time since fire was the most important predictor of nest survival (Yeldell, Cohen, Little, et al. 2017; Yeldell, Cohen, Prebyl, et al. 2017). Our site‐specific findings were likely driven by high canopy closure, where vegetation response to fire is minimal regardless of fire frequency, scale, season, or intensity (Jackson et al. 2007; Lashley et al. 2011). Although it was not our intent, the high canopy closure on our site decoupled time since fire from understory vegetation structure, demonstrating that there is nothing inherent to time since fire affecting nest success. This has important implications for turkey managers since the fire return interval needed to promote quality nesting cover will likely vary depending on site‐specific factors like canopy cover. We believe the overall low rates of artificial nest predation we observed were likely driven by low overall predator numbers, although we are uncertain to what extent our artificial nest study mimicked predation rates on actual turkey nests. Further research is needed to assess the indirect effects of large‐scale prescribed fire on turkey nest success. We also recommend that future studies examining turkey nest success include canopy cover as part of any vegetation assessments.

## Author Contributions

**Mariah G. McInnis:** data curation (lead), formal analysis (lead), investigation (lead), methodology (equal), writing – original draft (lead), writing – review and editing (equal). **Robert A. Gitzen:** conceptualization (supporting), data curation (supporting), formal analysis (equal), methodology (equal), project administration (supporting), supervision (supporting), writing – review and editing (equal). **Bret A. Collier:** formal analysis (supporting), methodology (supporting), project administration (supporting), writing – review and editing (supporting). **William D. Gulsby:** conceptualization (lead), data curation (supporting), formal analysis (supporting), funding acquisition (lead), investigation (lead), methodology (lead), project administration (lead), writing – review and editing (equal).

## Conflicts of Interest

The authors declare no conflicts of interest.

## Supporting information


Data S1.

## Data Availability

The datasets used and/or analyzed during the current study are available as supporting material in the file Data S1.
